# HER-2 low status in early-stage invasive lobular carcinoma of the breast: associated factors and outcomes in an institutional series

**DOI:** 10.1007/s10549-023-06927-x

**Published:** 2023-04-05

**Authors:** Harriet T. Rothschild, Elle Clelland, Anne Patterson, Julissa Molina-Vega, Mandeep Kaur, W. Fraser Symmans, Christopher J. Schwartz, A. Jo Chien, Rita A. Mukhtar

**Affiliations:** 1 School of Medicine, University of California, San Francisco, CA USA; 2 Department of Surgery, University of California, 1825 4th Street, 3rd Floor, Box 1710, San Francisco, CA 94143 USA; 3 Department of Pathology, MD Anderson Cancer Center, Houston, TX USA; 4 Department of Pathology, University of California, San Francisco, CA USA; 5 Department of Medicine, University of California, San Francisco, CA USA

**Keywords:** Invasive lobular carcinoma, HER2-low, Early-stage

## Abstract

**Purpose:**

HER2 overexpression has a central role in breast cancer carcinogenesis and is associated with poor prognosis if untreated. Lately, identification of HER2-low breast cancer has been proposed to select patients for novel HER2-directed chemotherapy and includes cancers with immunohistochemistry 1 + or 2 + with negative FISH, encompassing approximately 55–60% of all breast carcinomas. In early-stage breast cancer, the prognostic significance of HER2 low-disease is less well understood, with a particular paucity of data evaluating the prevalence and implications of HER2-low status in invasive lobular carcinoma (ILC).

**Methods:**

We evaluated 666 stage I-III ILC tumors from a prospectively maintained institutional database, comparing clinicopathologic features and disease-free survival (DFS) using a multivariable Cox proportional hazards model.

**Results:**

HER2-low status was common in this cohort of patients with ILC, but most clinicopathologic features did not differ between HER2-low and HER2-negative cases. However, when adjusting for tumor size, number of positive nodes, ER/PR status, and local therapy received, patients with HER2-low status had worse disease-free survival (DFS) than those with HER2-negative tumors (hazard ratio 2.0, 95% confidence interval 1.0–4.1, p = 0.05).

**Conclusion:**

This difference in DFS supports the notion that HER2-low and HER2-negative early stage ILC may differ clinically, despite similar clinicopathologic features. Further investigation into the potential benefit of HER2 targeted therapy in HER2-low early-stage breast cancer, and specifically lobular cancer, is warranted to ensure optimal outcomes in this distinct tumor subtype.

## Introduction

Human epidermal growth factor receptor 2 (HER2) is a family of transmembrane receptor tyrosine kinases encoded by the *ERBB2* gene, which has been an important area of research and treatment target in breast cancer [1]. Amplification of the *ERBB2* gene results in HER2 overexpression, which has a central role in promoting carcinogenesis in breast cancer and is associated with poor prognosis in untreated patients [2]. However, since the discovery of the monoclonal HER2 antibody trastuzumab, HER2 overexpression has predicted response to anti-HER2 treatment [3, 4]. Additionally, novel HER2-targeting antibody–drug conjugates (ADCs) have provided another way to potentially target HER2 in tumors that show low expression of HER2 [5]. A recent Phase 3 randomized trial demonstrated improved progression-free and overall survival for those with HER2-low disease treated with the ADC trastuzumab deruxtecan (NCT03734029/DESTINY-Breast04) in the metastatic setting [6].

Hence, there is new interest to define cancers with low levels of HER2 expression for this therapeutic approach, such as nonamplified (FISH-negative) tumors with immunohistochemistry (IHC) scores of 1 + or 2 + . By this definition, approximately 55–60% of breast carcinomas are HER2-low, of which 80% are hormone receptor positive and 15–20% are hormone receptor negative [1]. In clinical practice, these tumors are traditionally considered HER2-negative, since agents targeting biological dependence on the HER2 pathway, such as trastuzumab, have not been shown to offer clinical benefit [7]. In early-stage breast cancer, the prognostic significance of HER2 low-disease is less well understood, with a recent retrospective analysis showing no difference in outcomes by HER2-low status [5].

The prevalence and implications of HER2-low status in invasive lobular carcinoma (ILC) is currently unknown. ILC is the second most common type of breast cancer after invasive ductal carcinoma (IDC), representing 10–15% of all breast cancer. The majority of ILCs are hormone receptor (HR) positive and HER2-negative. Previous studies of HER2-low status in breast cancer consistently find a lower prevalence of HER2-low status in ILC compared to IDC [8–10]. To our knowledge, no studies have reported the prognostic significance of HER2-low status in early stage ILC.

In the current study, we aimed to describe the prevalence of HER2-low status in a mono-institutional cohort of early-stage ILC, with emphasis on clinicopathologic features and clinical outcome.

## Methods

With institutional review board approval, we retrieved clinicopathologic data from a prospectively maintained institutional database containing treatment and outcomes data for ILC patients undergoing surgery at the University of California, San Francisco between January 1996 and September 2019.

### Population

We included patients with tumors that had lobular or mixed lobular/ductal histology, and were stage I-III. HR positivity was defined as having ≥ 1% either estrogen receptor (ER) or progesterone receptor (PR) expression by immunohistochemistry. Both IHC and FISH testing for HER2 status were performed as part of standard clinical care on all cases. Tumors were classified as HER2-negative (IHC = 0), HER2-low (IHC = 1 + or 2 + with negative FISH [ratio of HER2/CEP17 < 2]), or HER2-positive (IHC = 3 + or FISH ratio ≥ 2).

### Clinicopathological parameters

The following baseline clinicopathological parameters were evaluated by HER2 status: age at diagnosis, menopausal status, body mass index (BMI), tumor stage, tumor histology, tumor grade, ER/PR status, molecular risk score (21-gene Recurrence Score or 70 gene signature when available), type of breast surgery, timing of chemotherapy administration (neoadjuvant or adjuvant). High 21-gene recurrence score (RS) risk was defined as including patients with a RS of 26 or greater, the intermediate-risk group as including patients with an RS between 11 and 25, and the low-risk group as including patients with an RS of 10 or less [12]. The 70 gene signature RS was categorized as high risk and low risk [13, 14]. Data were analyzed and reported using essential elements of REMARK criteria [15].

### Statistical analysis

The primary study end point was disease-free survival (DFS). DFS was defined as time from the date of primary surgery to the date of disease recurrence or death; patients alive without disease recurrence were censored at the date of last follow-up. Disease free survival was analyzed using the log rank test and Kaplan Meier method, and multivariate Cox proportional hazards models to estimate hazard ratios with 95% confidence intervals (CI) among HER2-negative and HER2-low cases with at least 6 months of follow up time and right-censored at 10 years. Data were analyzed in Stata 16.1 using chi-squared tests and t-tests. Data were analyzed between February 2022 and April 2022.

## Results

Six-hundred and sixty-six ILCs with available HER2 status were included in the analysis (Table 1). The mean age at diagnosis was 59.8 years (range 21–91), and the majority of patients were post-menopausal (69.3%). There were 408 (63.1%) patients with stage I disease, 160 (24.7%) with stage II, and 79 (12.2%) with stage III disease. Most tumors were grade 2 (n = 448, 68.5%), with 168 (25.7%) grade 1 and 38 (5.8%) being grade 3. The mean follow-up time was 6.7 years (standard deviation 5.4).

**Table 1:** Patient characteristics

Characteristics	HER2 negative (*n* = 184)	HER2-low (*n* = 434)	HER2 positive (*n* = 48)	*p-*Value
Age years, mean (SD)	60.2 (12.6)	59.6 (11.7)	61.2 (10.8)	0.63
Follow up time years, mean (SD)	6.5 (5.8)	6.6 (5.0)	8.1 (7.3)	0.19
Menopausal status				
Pre-menopausal	57 (32.6)	129 (31.1)	7 (17.9)	0.19
Post-menopausal	118 (67.4)	286 (68.9)	32 (82.1)	
BMI (kg/m^2^)				
≤ 24	96 (55.2)	209 (50.1)	23 (57.5)	0.73
25–30	45 (25.9)	123 (29.5)	11 (27.5)	
≥ 30	33 (18.9)	85 (20.4)	6 (15.0)	
Overall stage, n (%)				
I	113 (63.1)	263 (62.3)	32 (69.6)	0.66
II	41 (22.9)	108 (25.6)	11 (23.9)	
III	25 (14.0)	51 (12.1)	3 (6.5)	
Tumor grade				
1	55 (30.5)	107 (25.1)	6 (12.5)	0.09
2	118 (65.6)	292 (68.6)	38 (79.1)	
3	7 (3.9)	27 (6.3)	4 (8.4)	
Hormone receptor status				
ER +	179 (97.3)	420 (96.8)	43 (89.6)	0.03*
PR +	147 (79.9)	376 (86.6)	35 (72.9)	0.01*
Lympho-vascular invasion	8 (4.42)	23 (5.45)	6 (13.0)	0.07
Multifocal disease	58 (32.0)	133 (31.4)	11 (24.4)	0.59
Pleomorphic disease	18 (9.78)	43 (9.91)	16 (33.3)	< 0.001
Local treatment modality				
Lumpectomy	37 (20.4)	61 (14.2)	12 (25.5)	0.01*
Lumpectomy & radiation	74 (40.9)	139 (32.3)	14 (29.8)	
Mastectomy	44 (24.3)	166 (38.6)	15 (31.9)	
Mastectomy & radiation	26 (14.4)	64 (14.9)	6 (12.8)	

Data expressed as n (%) unless otherwise specified. Total *n* = 666

*HER2* Human Epithelial Growth Factor Receptor-2, *ILC* invasive lobular carcinoma, *ER* estrogen receptor, *PR* progesterone receptor

HER2-low status was common in the study cohort. Overall, 184 (27.6%) tumors were HER2-negative, 434 (65.1%) were HER2-low, and 48 (7.2%) were HER2-positive. HER2 status was associated with hormone receptor status, as HER2-low tumors were more likely to have PR positivity than both HER2-negative and HER2-positive tumors (86.6% compared to 79.9% of HER2-negative and 72.9% of HER2-positive tumors, p = 0.01). This difference remained significant when comparing HER2-low to HER2-negative tumors and excluding HER2-positive tumors (p = 0.034). However, there were no associations between HER2-low status and patient characteristics such as age, menopausal status, or BMI. Similarly, HER2-low status was not associated with tumor features including stage, grade, presence of lympho-vascular invasion, lobular carcinoma in situ, or multifocal disease. Within a subset of 304 HER2-negative or HER2-low tumors with either 70 gene signature results (n = 121) or 21-gene Recurrence Scores (n = 183) available, there was no association between HER2 status and molecular assay result.

HER2 positivity was significantly associated with a higher rate of pleomorphic ILC when compared to HER2-negative and HER2-low cases (33% versus 9.8% and 9.9% respectively, p > 0.001). Additionally, HER2-positive tumors were significantly less likely to be ER positive than both HER2-negative and HER2-low tumors (89.6% versus 97.3% and 96.8% respectively, p = 0.03).

Regarding treatment, we found no differences in use of neoadjuvant or adjuvant chemotherapy by HER2 status when comparing HER2-low to HER2-negative cases. However, patients with HER2-low tumors were more likely to undergo mastectomy versus lumpectomy when compared to HER2-negative and HER2-positive patients (53.7% versus 38.0% and 43.8% respectively, p = 0.001).

In unadjusted evaluation of DFS by the log rank test, there was no difference between HER2-negative and HER2-low cases. However, in a multivariable Cox proportional hazards model adjusting for tumor size, number of positive nodes, ER/PR status, and local therapy received, patients with HER2-low status had worse DFS than those with HER2-negative tumors (HR 2.0, 95% CI 1.0–4.1, p = 0.05) (Table 2). In this model, other factors associated with worse DFS included larger tumor size (HR 1.13, 95% CI 1.04–1.2, p = 0.005), increasing number of positive nodes (HR 1.1, 95% CI 1.1–1.13, p < 0.001), and undergoing lumpectomy without radiation for local therapy (HR 3.4, 95% CI 1.6–7.6, p = 0.002); PR positivity was associated with improved DFS compared to PR negativity (HR 0.4, 95% CI 0.2–0.8, p = 0.008).

**Table 2:** Multivariate Cox proportional hazards model for disease free survival in HER2-negative and HER2-low invasive lobular carcinoma (ILC) adjusting for local therapy, ER/PR status, tumor size, and number of positive nodes

	Disease free survival		
	Hazard ratio	95% CI	*P*
HER2 status			
Negative (ref)			
Low	2.02	1.0–4.07	0.05
Local therapy			
Lumpectomy + radiation (ref)			
Lumpectomy	3.45	1.56–7.63	0.002
Mastectomy	0.93	0.41–2.1	0.857
Mastectomy + radiation	1.01	0.39–2.62	0.982
Tumor size (per 1 cm)	1.13	1.04–1.24	0.005
Number of positive lymph nodes (per 1 node)	1.09	1.05–1.14	< 0.0001
ER status			
Negative (ref)			
Positive	0.49	0.17–1.4	0.193
PR status			
Negative (ref)			
Positive	0.41	0.21–0.79	0.008

Total *n* = 427

*HER2* Human Epithelial Growth Factor Receptor-2, *ER* estrogen receptor, *PR* progesterone receptor

## Discussion

In this study of 666 women with early-stage ILC, we found that the majority (65.1%) of tumors were HER2-low. Although two prior studies suggested that HER2-low positivity may be less likely in those with ILC [8, 9], the prevalence of HER2-low status in our cohort is similar to what is reported in hormone receptor positive breast cancer, irrespective of histology [1, 8, 16]. The prevalence of HER2-low ILC in our cohort supports a recently published study that compiled data from various datasets and included roughly 500 patients with ILC [17]. By contrast, a single institution retrospective review with fewer patients found a higher prevalence of HER2-low ILC compared to IDC [18].

We found very few differences in clinicopathologic features between HER2-low and HER2-negative disease. Interestingly, we found that that those with HER2-low disease were significantly more likely to have PR positive tumors than those with either HER2-negative or HER2-positive disease. Since PR positivity is usually associated with improved prognosis, the significance of this finding is unclear, especially since we found that HER2-low cases in this cohort had modestly but significantly worse DFS that HER2-negative cases. This difference in PR status may reflect other underlying biologic differences between HER2-low and HER2-negative ILC which needs further investigation. In our cohort, HER2-low ILC patients were more likely to have had a mastectomy versus lumpectomy, while a recent large study of early-stage breast cancer showed no association between type of breast surgery and HER2 status [11]. The underlying cause for increased mastectomies in HER2-low patients in our cohort is unclear, as we did not find an association between tumor size, age, or multifocality and HER2 status.

The current literature provides mixed results regarding the prognostic significance of HER2-low status in early-stage breast cancer show. Two large retrospective studies from Japan and Korea found no difference in overall survival between early-stage HR positive HER2-low and HER2-negative patients [10, 18] On the other hand, a recently published study of early-stage HR positive breast cancer found that while HER2-low status was not associated with recurrence free survival overall, there was a trend towards increased risk of recurrence specifically among those with lobular histology and HER2-low disease [8]. In our analysis, we similarly found an association between HER2-low status and worse outcomes in those with ILC when adjusting for local therapy, hormone receptor status, tumor size, and number of positive nodes. Our findings combined with the previously reported trend suggest that HER2-low status should be explored further in early-stage breast cancer, and ILC specifically. This is particularly relevant given the recent reports of clinical activity using the ADCs in HER2-low metastatic breast cancers, and the possibility that some strategies of targeting HER2 might be effective in early-stage HER2-low disease as well [19, 20].

One limitation of this study and others is that HER2 IHC interpretation is observer dependent. In our institution, HER2 IHC is reviewed by subspecialized breast pathologists as part of routine clinical care, and cases undergo reflex FISH testing, irrespective of IHC score. Currently, there remains no assay to reliably identify HER2-low breast cancers. Another limitation is that this data is a retrospective study from a single cancer center, and thus may not be representative of a larger population. Additionally, retrospective analyses are subject to treatment selection bias.

However, our findings support the notion that HER2-low and HER2-negative disease may represent two different clinical entities within ILC. It should be noted that there is an important distinction between molecular classification based on biological drivers (discriminants of the disease itself) and the drug targets designed into novel ADCs. Given the recent finding of therapeutic benefit of ADCs in HER2-low breast cancer in the metastatic setting, identifying potential tumor types that might derive benefit of such approaches in the early-stage setting is of clinical interest. Further investigation of the potential benefit of HER2 targeted therapy in ILC, which predominately lacks HER2-amplified disease, is warranted to ensure optimal outcomes in this understudied tumor subtype currently treated similarly to the more common ductal cancers.

## Conclusions

Overall, our findings illustrate a high prevalence of HER2-low disease in early stage ILC. Further studies are required to better understand HER2-low breast cancer in general, and in particular in ILC, the second most common type of breast cancer.

## Data Availability

The data supporting all tables in this published article are not publicly available to protect patient privacy, but can be accessed from the corresponding author on request. Data will be made available to authorized researchers who have obtained Institutional Review Board (IRB) approval from their own institution and from the University of California, San Francisco IRB.

## Abbreviations

HER2: Human epidermal growth factor receptor 2
ADC: Antibody-drug conjugates
FISH: Fluorescent in situ hybridization
ILC: Invasive lobular carcinoma
IDC: Invasive ductal carcinoma
HR: Hormone receptor
IHC: Immunohistochemistry
ER: Estrogen receptor
PR: Progesterone receptor
BMI: Body mass index
RS: Recurrence score
DFS: Disease free survival
